# Spatio-temporal expression of ANK2 promotes cytokinesis in oocytes

**DOI:** 10.1038/s41598-019-49483-5

**Published:** 2019-09-11

**Authors:** Anna Tetkova, Denisa Jansova, Andrej Susor

**Affiliations:** 1 Laboratory of Biochemistry and Molecular Biology of Germ Cells, IAPG CAS, Libechov, Czech Republic; 2 Department of Cell Biology, Faculty of Science, Charles University, Prague, Czech Republic

**Keywords:** Meiosis, Oogenesis

## Abstract

In the absence of transcription, the regulation of gene expression in oocytes is controlled almost exclusively at the level of transcriptome and proteome stabilization, and translation. A subset of maternal transcripts is stored in a translationally dormant state in the oocyte, and temporally driven translation of specific mRNAs propel meiotic progression, oocyte-to-embryo transition and early embryo development. We identified *Ank2.3* as the only transcript variant present in the mouse oocyte and discovered that it is translated after nuclear envelope breakdown. Here we show that *Ank2.3* mRNA is localized in higher concentration in the oocyte nucleoplasm and, after nuclear envelope breakdown, in the newly forming spindle where its translation occurs. Furthermore, we reveal that *Ank2.3* mRNA contains an oligo-pyrimidine motif at 5′UTR that predetermines its translation through a cap-dependent pathway. Lastly, we show that prevention of ANK2 translation leads to abnormalities in oocyte cytokinesis.

## Introduction

When oocytes reach their full size they undergo striking changes in nuclear morphology due to large-scale chromatin condensation leading to transcriptional silence^1^. In the absence of RNA synthesis, completion of meiosis and early embryonic development rely on maternally synthesized mRNAs^2^. Highly stable maternal RNAs^3,4^, stored prior to meiotic reentry in the oocyte growth period, drive the meiotic progression of the oocyte and early embryo^5–7^. Thus, the regulation of gene expression in oocytes during the growth period is controlled at the level of stored mRNA translation and any mRNA metabolism would have a significant effect at this stage of development.

In addition to qualitative and quantitative control of protein levels, determining when and where the proteins are produced within the cell plays a role in the cell physiology^8,9^. The appropriate localization of mRNA within a cell is an essential requirement for the correct propagation of genetic information, as well as being an efficient way to regulate cellular processes. In many species, including fly and frog, the differential localization of protein synthesis is achieved by distribution of mRNAs^10–12^. This is critical for the determination of the animal and vegetal poles in frog oocytes, which requires the proper asymmetric distribution of several mRNAs^13^. However, little is known about the patterning of mammalian oocytes. Although many mRNAs localize randomly throughout the cytoplasm, some are highly accumulated in distinct cellular areas. The nucleus of the mouse oocyte demonstrates a significant contribution to the RNA distribution of the intercellular patterning^14–17^. However, the proteins which need to be synthesized, and their spatio-temporal regulation of translation in the oocytes, have not yet been studied.

Here, we describe the regulation of the spatio-temporal translation of the specific mRNA *Ank2.3* in the mouse oocyte. We show that the cell develops mechanisms to retain such specific mRNA in the nucleus to ensure its spatio-temporal expression in the newly forming spindle, which modulate mammalian oocyte cytogenetic events.

## Results

### ***Ankyrin 2.3*is oocyte specific mRNA**

**Figure 1:**
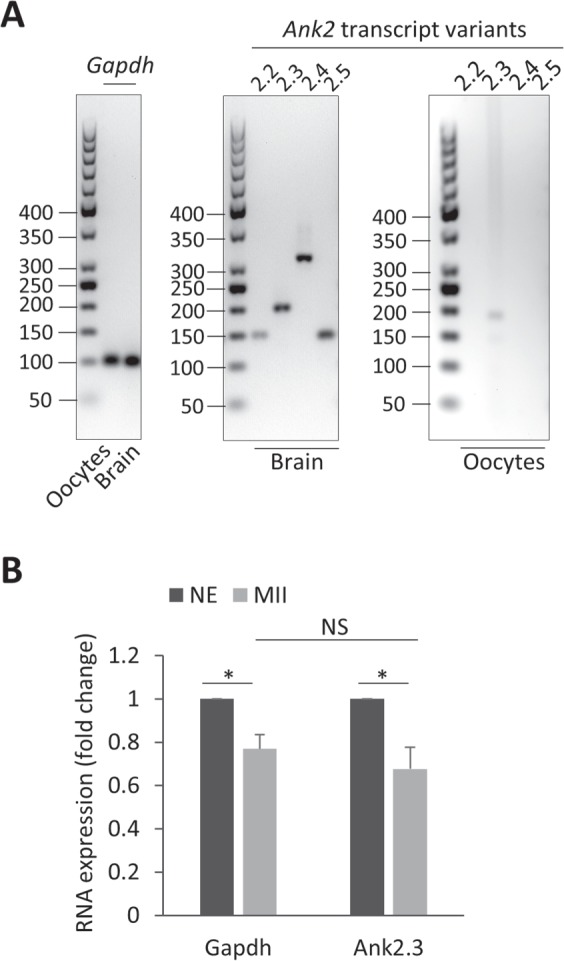
*Ank2.3* is an oocyte specific transcript variant stably expressed during meiotic maturation. (**A**) PCR analysis of four transcript variants of *Ank2* mRNA (2.2, 2.3, 2.4 and 2.5). Occurrence of transcript variants in the brain and oocytes from mouse RNA. *Gapdh* mRNA was used as a cDNA loading control. Representative images from at least three independent replicates. (**B**) qRT-PCR mRNA expression of *Gapdh* and *Ank2.3* in the NE (nuclear envelope containing oocytes before meiotic maturation) and MII (metaphase II) oocytes. Data from three independent experiments were normalized to NE oocytes and *Ank2.3* to the internal standard *Gapdh*. Fold change, mean, error bars are SD; Student’s *t*-test, *P < 0.05; NS non-significant. See also Suppl. Figs 1, 2 and 5.

Ankyrin 2 (ANK2) protein is an essential component of the cell cytoskeleton and is abundant in the brain^18,19^. By PCR, we found that despite all four transcript variants of *Ank2* being expressed in the mouse brain, only one variant, *Ank2.3* mRNA, is present in the oocyte (Fig. 1A). Additionally, we performed RNA FISH to determine the presence or absence of *Ank2.3* mRNA within the oocyte and within cumulus cells (a different type of cell that surrounds the oocyte). The oocyte contained a significant amount of *Ank2.3* mRNA foci (Suppl. Fig. 1A,B) however, the RNA signal in the cumulus cells (Suppl. Fig. 1A,B) was comparable to the negative control *DapB* RNA (Suppl. Fig. 1C,D) which is absent in eukaryotic cells^20,21^. To analyze mRNA stability during oocyte meiotic progression we performed qRT-PCR using *Gapdh* as a reference transcript. Both *mRNAs* showed a slight decrease (~25%; *P < 0.05) from NE (oocytes containing nuclear envelope, prior to the meiosis onset) to MII (metaphase II) transition (Fig. 1B). Contrastingly, the measurement of *Neat2* lncRNA revealed a dramatic decrease (75 ± 2%; ***P < 0.001) in NE to MII oocytes (Suppl. Fig. 2A).

We found that the oocyte transcript variant *Ank2.3* persists during oocyte meiotic progression from the NE to MII transition.

### *Ank2.3* mRNA is abundant in the oocyte nucleus and in the newly forming spindle

**Figure 2:**
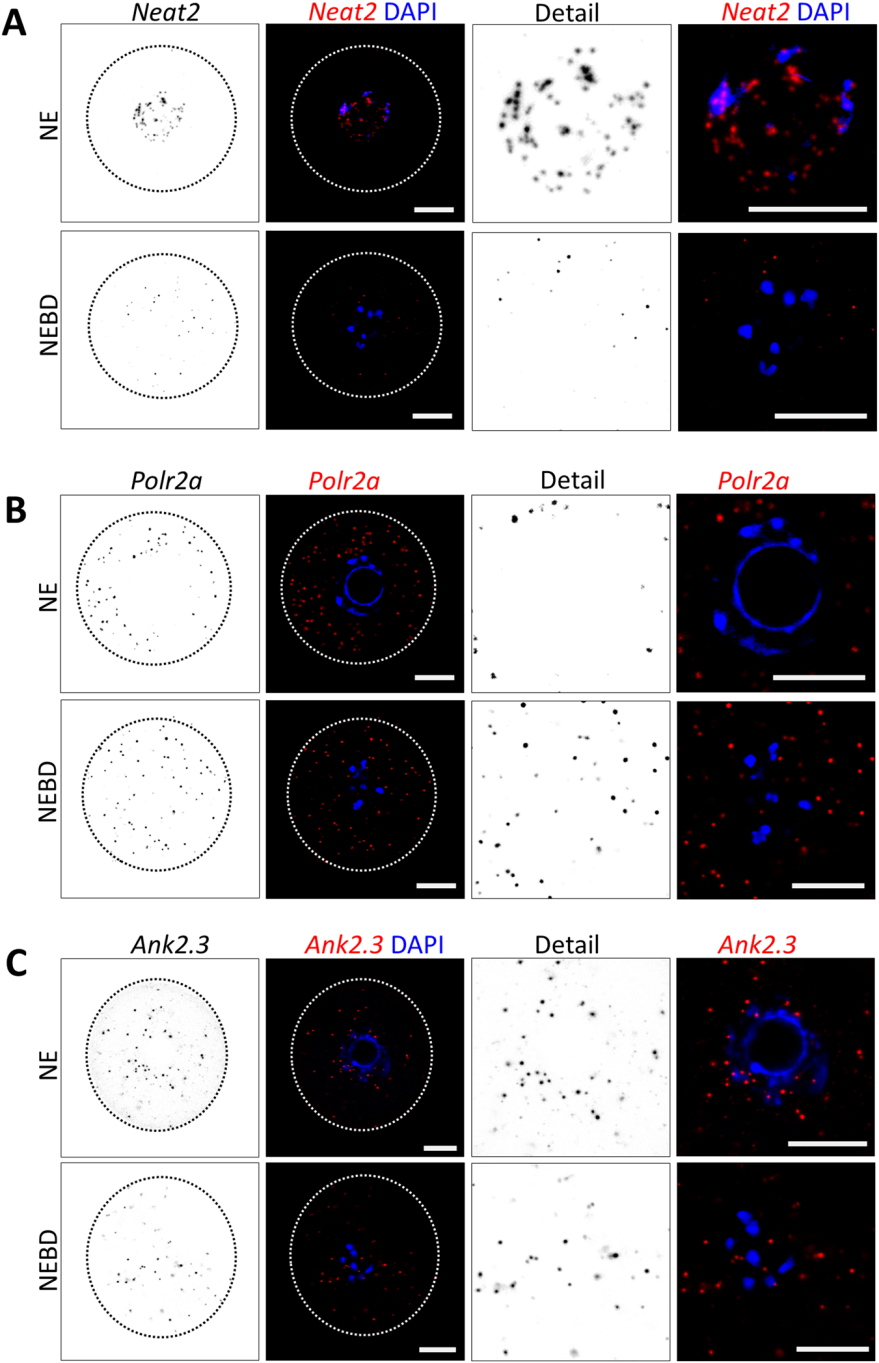
Localization of lncRNA *Neat2* and mRNAs coding for *Polr2a* and *Ank2.3* in NE and NEBD oocytes. Single Z-scan confocal images from RNA FISH for (**A**) *Neat2* RNA (**B**) *Polr2a* mRNA and (**C**) *Ank2.3* mRNA in NE (0 h) and NEBD (nuclear envelope breakdown; 3 h) oocytes. RNA in grey and red and DNA in blue (DAPI). The cortex of the cell is depicted by a black or white dotted line. Representative images from at least three independent experiments (n ≥ 18), scale bar = 20 μm. Bacterial *DapB* RNA (Bacillus subtilis, str. SMY; EF191515.1) was used as a negative control. See also Suppl. Fig. 1C,D.

**Figure 3:**
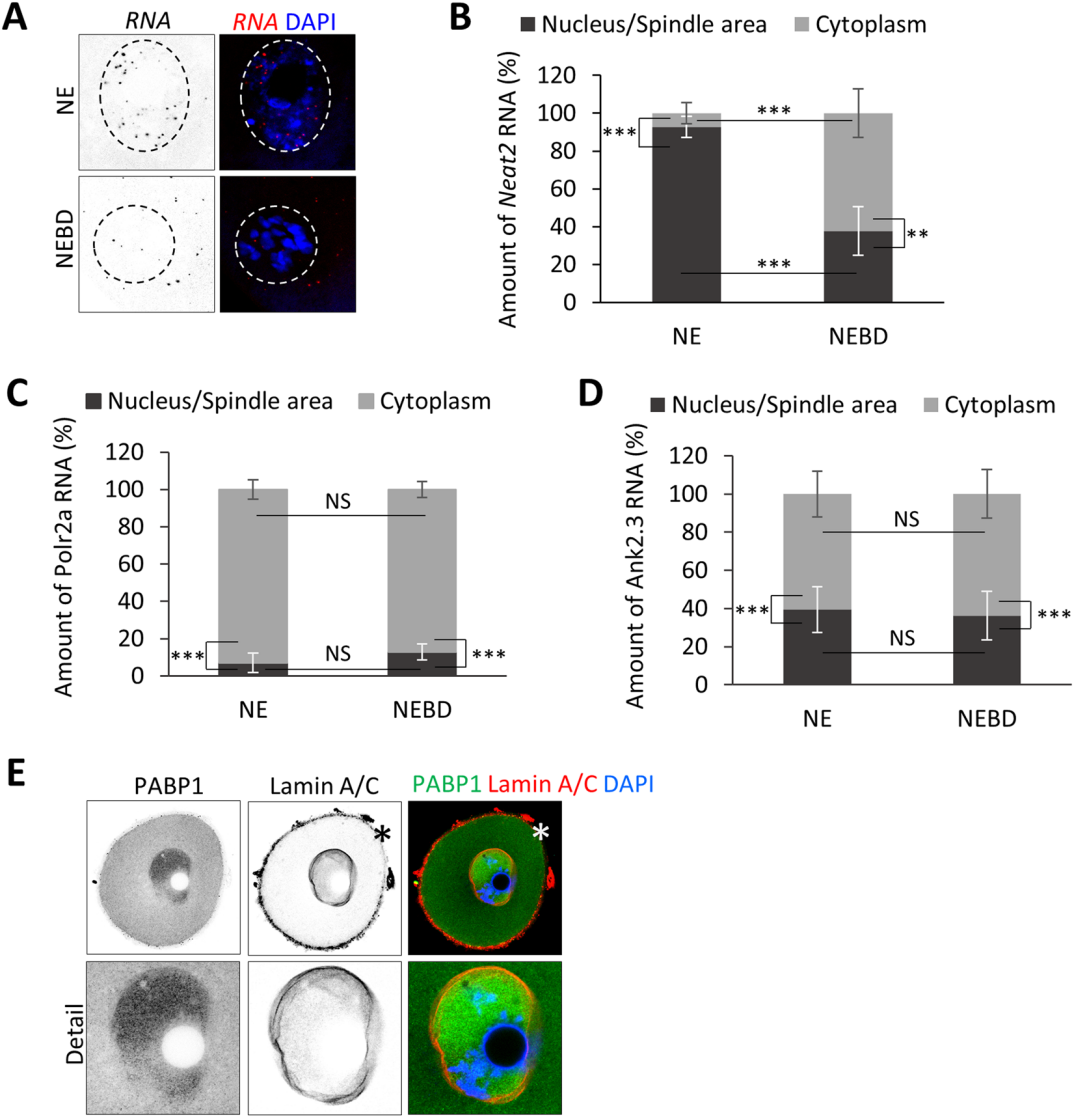
Quantification of RNA localization in the nucleus, spindle area and cytoplasm. (**A**) Scheme of RNA foci counting in the nucleus and spindle area (black) and cytoplasm (grey) in NE and NEBD oocytes. Dashed line depicts nuclear/spindle area. (**B**) Quantification of confocal images from RNA FISH of RNA coding for *Neat2*, (**C**) *Polr2a*, and (**D**) *Ank2.3*. Mean, error bars are SD; (n ≥ 18), Student’s *t*-test; **P < 0.01; ***P < 0.001; NS non-significant. Bacterial *DapB* RNA was used as a negative control. See also Suppl. Fig. 1C,D and 2. (**E**) Representative confocal images of localization of PABP1 (green) in the oocyte. LMN A/C (red) was used for visualization of nuclear membrane, asterisk denotes nonspecific antibody binding to zona pellucida. DNA in blue.

We examined the localization of *Ank2.3* mRNA in the oocyte. As controls we used RNA candidates with known intracellular localization; nuclear lncRNA *Neat2*^22^, cytoplasmic *Polr2a* mRNA^23,24^ and negative control *DapB* RNA. By RNA FISH we detected the transcript distribution in the NE and NEBD (post nuclear envelope breakdown, 3 h) stages of oocytes. As expected *Neat2* displayed nuclear localization (Fig. 2A), *Polr2a* cytoplasmic localization (Fig. 2B) and interestingly *Ank2.3* mRNA was found in both the nucleus and the cytoplasm (Fig. 2C). Quantification of RNA foci in the nucleus and in the newly forming spindle (vicinity of chromosomes; Fig. 3A) indicates that *Neat2* RNA is localized almost exclusively (93 ± 5.6%) in the nucleus of oocyte (Fig. 3B) and by 38% (±12.9%) in the spindle area of post NEBD (Fig. 3B). Despite equal total expression of both candidates *Polr2a* and *Ank2.3* (P > 0.05; Suppl. Fig. 2B), *Polr2a* mRNA was significantly less (7 ± 5.2%) in the nucleoplasm (Fig. 3C) with the lowest presence in the vicinity of the chromosomes (12.8 ± 4.3%; Fig. 3C). *Ank2.3* mRNA was abundant (39.5 ± 12%) in the nucleoplasm (Fig. 3D) as well as in the newly forming spindle (36.3 ± 12.7%; Fig. 3D). *DapB* RNA showed no RNA foci (Suppl. Fig. 1C,D). Expression of candidate RNAs, which we observed in NE and NEBD oocytes revealed a stable level of *Ank2.3* and *Polr2a* (Suppl. Fig. 2B), while *Neat2* was significantly reduced from post NEBD to MII (Suppl. Fig. 2A,B).

3′UTR might influence RNA localization^25^ in the cell thus we examined 3′UTR elements which might have role in transcript localization and translation. We searched specifically for nucleocytoplasmic shuttling protein Poly(A) Binding Protein (PABP1)^26–28^ elements (UGUGUA or AUAUAUA)^26^. We found that three PABP1 elements are present at the 3′UTR of *Ank2.3* mRNA and in the *Neat2* lncRNA, however none in the 3′UTR of *Polr2a* mRNA. Next we performed ICC to test if the PABP1 protein is localized in the nucleoplasm of the oocyte. We found that PABP1 protein is enriched in the nucleoplasm (Fig. 3E) of the NE oocyte. *Ank2.3* 3′UTR also contains two polyadenylation signals (PAS) and four cytoplasmic polyadenylation elements (CPE) on the other hand *Polr2a* only single PAS which might influence their translation post NEBD^29^.

Our results indicate that the presence of a transcript in the nucleoplasm links their consequent localization to the spindle assembly area of the newly forming spindle. Moreover, our candidate mRNAs *Ank2.3* and *Polr2a* appear to be stable post NEBD, compared with *Neat2* RNA which had dramatically decreased levels. Additionally our results indicate that PABP1 is localized in the nucleus and our candidate RNAs localized in the nucleus contains PABP1 biding elements which might be prerequisite for their localization and fate in the oocyte.

### *Ank2.3* is translated in the newly forming spindle

**Figure 4:**
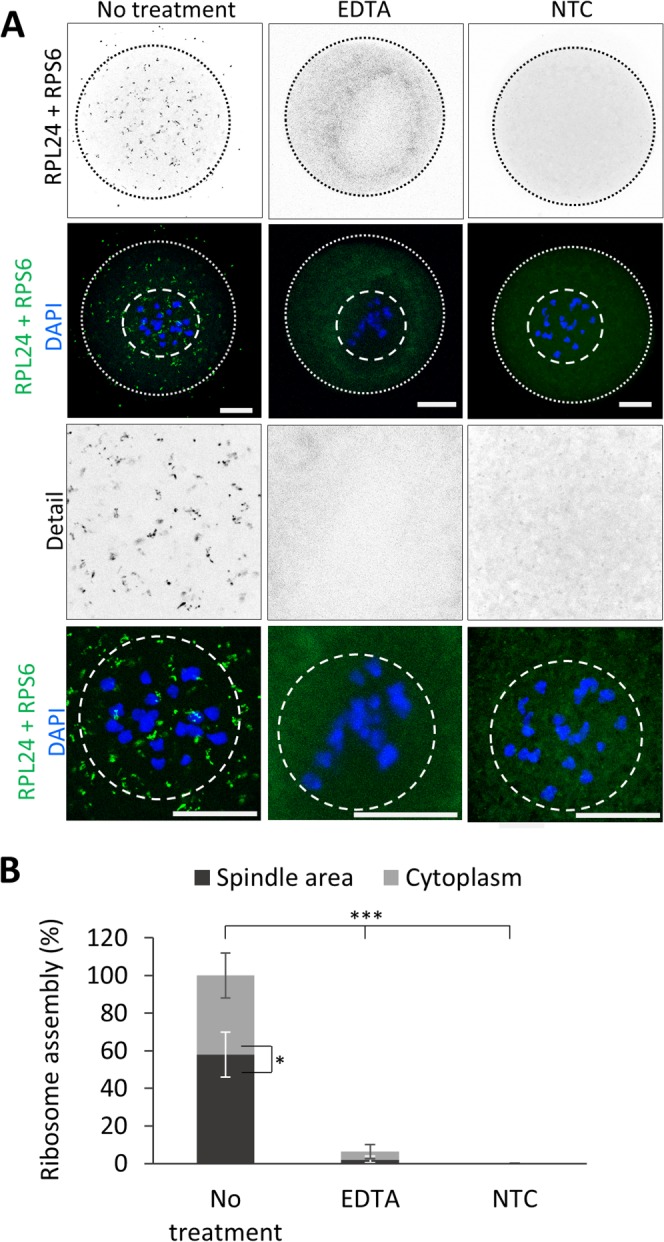
Spindle area contains assembled ribosomes. (**A**) Proximity ligation assay (PLA) detecting ribosome assembly using RPL24 and RPS6 markers (grey and green dots). EDTA was used for ribosome disruption. As a negative control (NTC) RPS6 antibody was omitted. The cortex of the oocytes is depicted by a black or white dotted line; dashed line shows spindle area. Representative images of three independent experiments (n ≥ 15); scale bar = 20 μm. (**B**) Quantification of ribosome assembly events in NEBD oocytes. Mean; error bars are SD; Student’s *t*-test, *P < 0.05; ***P < 0.001.

Localization of an mRNA might be a prerequisite for its translation and protein function^9,10,30^. First, we performed a proximity ligation assay (PLA) to detect localization of assembled ribosomes in NEBD oocytes (Fig. 4A). For detection of localization of ribosome assembly in the cell, ribosomal markers RPL24 and RPS6 were used^31^. We found that assembled/translating ribosomes are distributed evenly in the cytoplasm. Measurements of the spindle assembly area show presence of ribosome assembly signal (Fig. 4A,B). EDTA treatment was used for disruption of ribosomes which shows disappearance of interaction of RPL24 with RPS6. Similarly omission of RPS6 antibody in our negative technical control (NTC) visibly reduced ribosome interaction (Fig. 4A,B).

**Figure 5:**
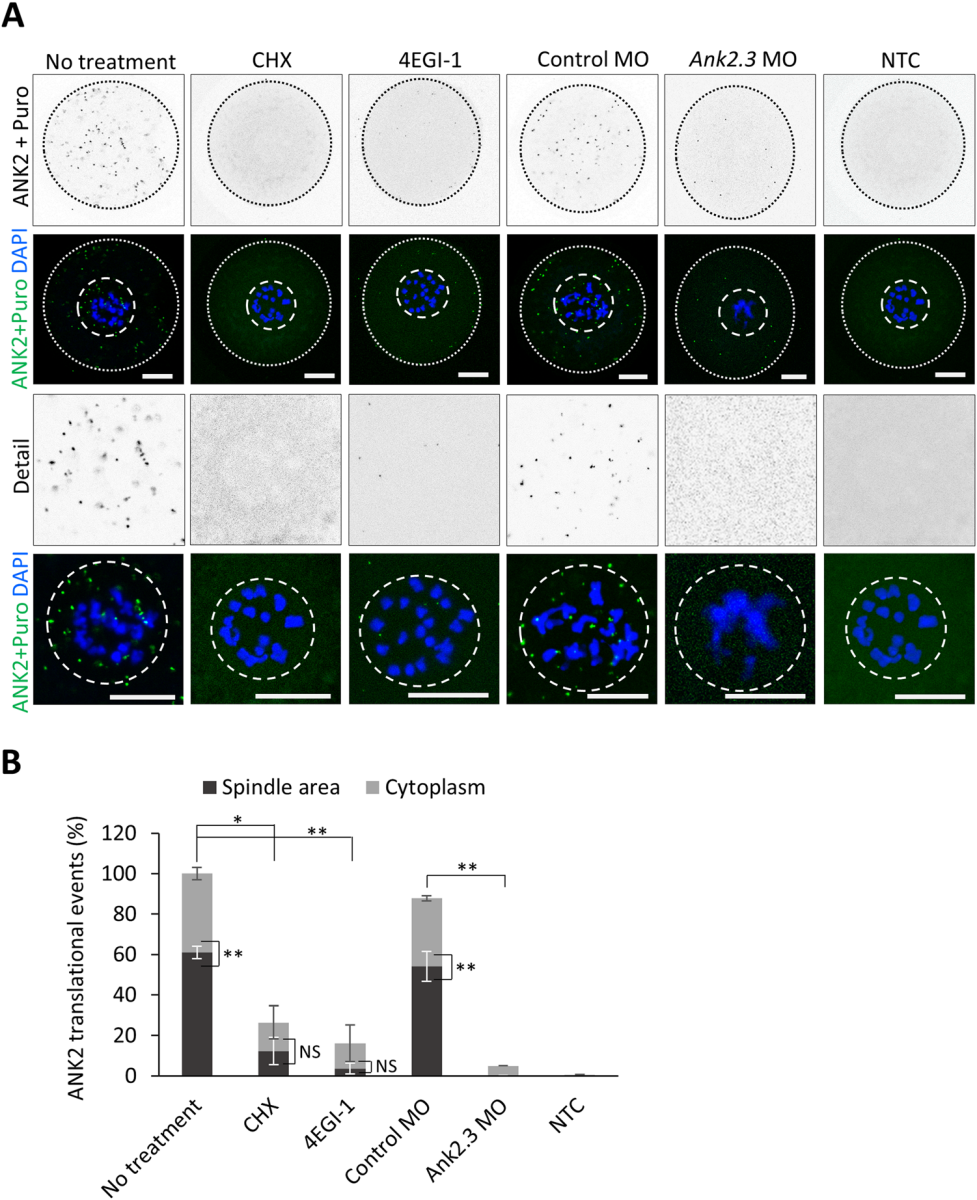
*ANK2* is translated at the newly forming spindle through cap-dependent translation. (**A**) Ribopuromycilation combined with Proximity ligation assay (Puro-PLA) were performed on NEBD stage oocytes in the absence or presence of cycloheximide (CHX) and eIF4E/eIF4G1 interaction inhibitor (4EGI-1). Microinjection of morpholinos (MO) was used to specifically suppress *Ank2.3* mRNA translation in the oocyte. ANK2 translational events in grey and green scales, DNA in blue. The cortex of the cell is depicted by a black or white dotted line; dashed line shows spindle area. Representative images from at least three independent experiments (n ≥ 15), scale bar = 20 μm. As a negative control (NTC) ANK2 antibody was omitted. (**B**) Quantification of ANK2 translational events at the spindle area (black) and cytoplasm (grey). Mean, error bars are SD; Student’s *t*-test; *P < 0.05; **P < 0.01; ***P < 0.001; NS non-significant. See also Fig. 6D and Suppl. Figs 3 and 5.

**Figure 6:**
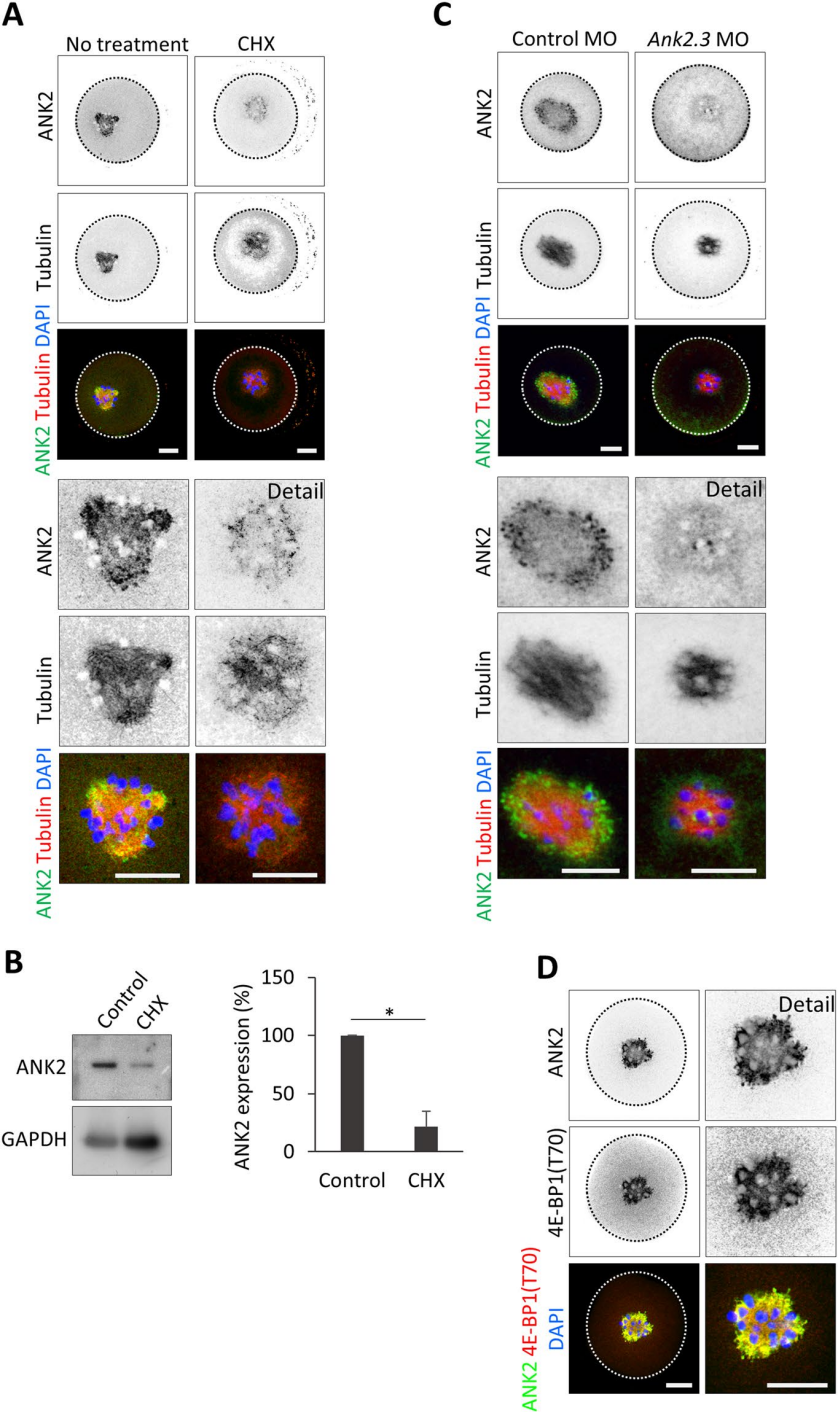
ANK2 protein translated post NEBD and localized in the spindle together with inactivated cap-dependent translational repressor 4E-BP1. (**A**) Representative confocal images of NEBD oocytes labeled with ANK2 and tubulin antibodies in absence or presence of CHX. N ≥ 20; scale bars 20 µm. Details of spindle areas are below. For localization of ANK2 protein during meiotic progression see Suppl. Fig. 4. (**B**) Representative immunoblot images detecting ANK2 and loading control GAPDH in oocytes treated with CHX. Graph shows quantification of immunoblots probed for ANK2 and loading control GAPDH antibody. Data from three independent experiments; Student’s *t*-test, *P < 0.05; mean SD ± 13.19). (**C**) Microinjection of morpholinos suppress ANK2 expression at the newly forming spindle. ANK2 (green), tubulin (red) and DAPI (blue). Scale bar 20 µm. Details of spindle areas are below. D) Representative confocal images of NEBD oocytes immunolabeled by ANK2 (green) and phosphorylated 4E-BP1(Thr70) (red) antibodies, DNA in blue (DAPI). From two independent biological replicates, n = 30. Average Pearson’s coefficient = 0,71 (SD ± 0,04); average colocalization rate (=colocalization area/area foreground) = 91, 21% (SD ± 6.47).

Next we performed ribopuromycilation with PLA (Puro-PLA)^32^ to detect specific translational events of our candidate *Ank2.3* mRNA in the NEBD oocytes (Fig. 5A) and found that the majority of these events occur in the spindle area (60 ± 3.1%) (Fig. 5A,B). Treatment by CHX significantly reduced the translation of *Ank2.3* in the oocyte (Fig. 5A,B) with a visible decrease in the newly forming spindle (Fig. 5A,B). *Ank2.3* mRNA has a terminal oligo-pyrimidine (TOP) RNA motif^33,34^; Suppl. Fig. 3, suggesting a cap-dependent mechanism for its translation^35^. Indeed, the suppressor of the eIF4F complex formation, 4EGI-1^36^, significantly decreased ANK2 translational events (Fig. 5A,B). Microinjection of control morpholinos (MO) does not influence ANK2 however MO inhibiting translation of *Ank2.3* mRNA results in the significant decrease of translational events in the cell (Fig. 5A,B). Similarly, omitting the ANK2 antibody significantly decreased the PLA signal (NTC; Fig. 5A,B). In connection to our previous results we analyzed ANK2 protein expression and localization in the oocytes using immunocytochemistry (ICC) and immunoblot. Labeling ANK2 and spindle marker tubulin in the NEBD oocyte revealed the presence of ANK2 protein in the forming spindle (Fig. 6A,C). CHX treatment and repression of *Ank2.3* mRNA translation by microinjection of morpholinos suppressed the expression of ANK2 in the oocyte and in the spindle (Fig. 6A–C). Furthermore, staining of major meiotic stages of oocyte development: NE (0 h), NEBD (3 h), metaphase I (MI; 7 h) and metaphase II (MII; 12 h) showed a gradual increase of ANK2 protein in post NEBD with clear localization to newly developing spindle or to fully formed bipolar spindle (Suppl. Fig. 4A). In detail, ANK2 protein is localized with polymerized tubulin on the spindle with cytoplasmic protrusions (Suppl. Fig. 4B).

The results above indicate that *Ank2.3* is translated by a cap-dependent mechanism, therefore, we additionally performed ICC with inactivated repressor of cap-dependent translation 4E-BP1^37^. We detected a high colocalization rate of ANK2 protein with 4E-BP1(Thr70); average Pearson’s coef. = 0.71 (±0.04), average colocalization rate = 91.21% (±6.47%) in the newly forming spindle (Fig. 6D).

We observed through multiple approaches the spatio-temporal translational process for the specific protein ANK2. *Ank2.3* mRNA was translated by a cap-dependent mechanism during the meiotic onset in the spindle forming area.

### ANK2.3 is essential for oocyte cytokinesis

**Figure 7:**
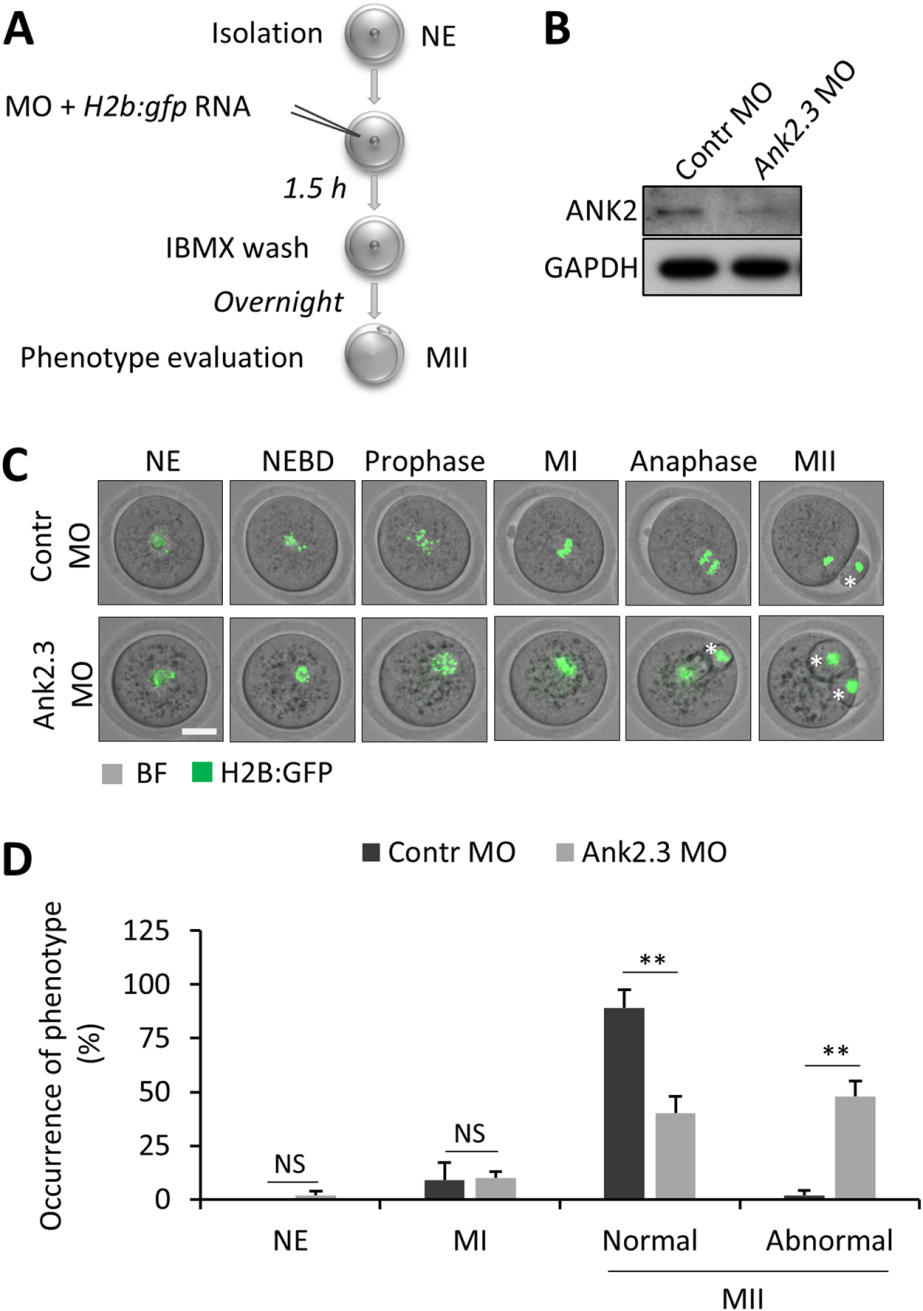
Downregulation of ANK2 results in defects in oocyte meiosis. (**A**) Experimental scheme of downregulation of ANK2 in the oocyte. (**B**) Expression of ANK2 protein is decreased after microinjection of morpholinos (MO) against *Ank2.3* mRNA. GAPDH was used as a loading control. Representative image from three independent experiments. (**C**) Morphology of the meiotic progression of oocytes microinjected with Control and *Ank2.3* morpholinos (MO). RNA coding for H2B:GFP was co-injected with morpholinos to track chromosomes. Asterisk depicts polar body, scale bar 30 µm. (**D**) Quantification of abnormal phenotypes (aberrant spindle formation and cytokinesis – polar body extrusion) in the morpholino microinjected oocytes. From three independent biological experiments (n ≥ 31). Presented as mean ± SD, Student’s *t*-test **p < 0.01, NS non-significant. See also Suppl. Fig. 5.

To determine the role of ANK2 expression in oocyte development we used microinjection of morpholinos (MO)^38^ to block the translation of *Ank2.3* in the oocyte (Fig. 7A). Control MO (Contr MO) or morpholinos targeting the translation of *Ank2.3* mRNA (Ank2.3 MO) were injected into NE oocytes together with chromosome marker histone 2B tagged with GFP (*H2b:gfp)* RNA (Fig. 7A). Immunoblot analysis showed that MO efficiently suppresses translation of ANK2 in the oocyte (Fig. 6B). Next, we performed time lapse microscopy of meiotic maturation of the injected oocytes (Fig. 7C) which revealed that there are no differences in the meiotic progression of NEBD but there are significant abnormalities (P < 0.01) in oocyte cytokinesis (first polar body extrusion) (Fig. 7C,D). Oocytes with decreased expression of ANK2 displayed an altered phenotype when 48% (±7%; n ≥ 31) of oocytes extruded whole genetic information in two polar bodies (Fig. 7C).

Here we found that synthesis of ANK2 protein is required for normal oocyte cytokinesis and promotes genomic stability in the mammalian oocyte.

## Discussion

We present here a mechanism for cell cycle-regulated protein expression of candidate mRNA which is essential for normal mammalian oocyte development. The essentiality of proteosynthesis for meiotic and mitotic progression has been characterized previously^39,40^. Studies revealed that although protein synthesis is not essential for NEBD in mouse oocytes, it is required for the spindle assembly and the progression to metaphase II^39^. During transcriptional silence with a deposit of maternally synthesized RNAs, translation becomes a key factor to drive M-phase progression^40^. Here we describe a mechanism of translational regulation of *Ank2.3*, a mouse oocyte only transcript variant of *Ankyrin 2* mRNA. Previously, we and others reported that the nucleus accommodate an enriched poly(A) RNA population^14,15,41^ and specific mRNA^17,42–44^ which might contribute to the translational activity in the newly forming spindle post NEBD. However, the identity of the proteins needed to be synthesized and their spatio-temporal regulation of translation in oocytes had not been previously studied.

*Ank2.3* is the only transcript variant present in the oocyte and contains a 5′-teminal oligopyrimidine tract (TOP) which leads to its translation through a cap-dependent translation pathway^45,46^. This utilization is in line with the observed activity of mTOR–eIF4F on newly developing spindle^14,47–50^. *Ank2.3* mRNA demonstrated nucleoplasm enriched localization with a significantly higher occurrence than *Polr2a* mRNA which was excluded from the nucleoplasm in the interphase cell^23^. Here we found that binding elements for nucleocytoplasmic shuttling RNA binding protein PABP1 are present at 3′UTR of *Ank2.3* mRNA and in the lncRNA *Neat2*. Presence of PABP1 in the nucleoplasm of oocyte might be a step in specific RNA localization providing an opportunity for coordinated control of specific RNA localization and possibly translation post NEBD.

We propose that RNA localization into the nucleus leads to its stabilization and/or translational dormancy. Such stabilization of RNA in the nucleus might be applied to *Neat2* RNA that is almost exclusively localized into the nucleus of oocytes and subsequently reduced during nuclear envelope breakdown. Furthermore, localization of RNA into the nucleus might be a mechanism to maintain translational dormancy of specific mRNA (*Ank2.3*). The translational repressor 4E-BP1 accumulated in the nucleus^15,47,51^, possibly due to mRNA import from the cytoplasm. Moreover, the nucleoplasm contained eIF4A3, an exon junction complex protein which is deposited onto mRNAs and released during the pioneer round of translation^52,53^ which follows NEBD. The nuclear localization of both 4E-BP1 and eIF4A3 points to the posttranscriptional translational silencing of *Ank2.3*. Previously, it has been shown that the repressive activity of 4E-BP1 becomes significantly decreased post NEBD due to the spatio-temporal nature of the spindle assembly area via mTOR–eIF4F axis^14,46,47,54–57^. In this context, the spatio-temporal activity of the mTOR–eIF4F axis is coupled with the temporal activity of the MPF. CDK1-CyclinB showed spatio-temporal activation, which influences the mTOR–eIF4F axis in the oocyte^47,58,59^. This offers an elegant explanation for the regulatory mechanism of the dormant maternal transcripts in the appearance and timing in oocytes of different species. Active localization and translation of specific mRNAs in *X. leavis* meiotic spindles have previously been observed^60^, fully consistent with our findings. mRNAs were also detected in the more advanced MII spindles of mouse oocytes^55,61^. In addition to mRNA localization, translational initiation factors, assembled ribosomes and RNA-binding proteins are present in the mitotic or meiotic spindle (our study^7,60,62^). One possible explanation of spatio-temporal differences in RNA retention in the spindle region might be through an organelle exclusion membrane system which causes molecular crowding in sufficient amounts to influence the accumulation of nuclear RNAs post NEBD^14,63^. To our knowledge, cytoplasmic flow in the mouse oocyte becomes evident after the spindle becomes bipolar and asymmetric spindle positioning occurs^64^ in the later stages of meiosis^65^. Actin is a key player of spindle positioning and is a cytokinesis factor^66–68^. ANK2 has a known role in linking the actin cytoskeleton to integral membrane proteins^69,70^ which could lead to the observed aberrant cytokinesis after interrupting its *de novo* translation. Similarly, downregulation of the ANK2 homolog in *C. elegans* displayed defects in germline cytokinesis resulting in sterility^71^.

The three conserved molecular modules for M-phase progression, mRNA localization, raising of MPF and mTOR–eIF4F activity post NEBD, may be present in other species, including humans, due to similar spatio-temporal localization and activation of the key components^15,47,59^. Therefore, other organisms may also share the behavior we described here. In conclusion, we have found that an oocyte develops a mechanism to retain a specific subset of mRNAs in its nucleus which predetermines RNA localization and translation on the meiotic spindle contributing to highly asymmetric division. The next challenge is to identify the pool of nucleoplasm enriched transcripts and the mechanisms that mediate localization in the nucleus.

## Methods

### Oocyte isolation, cultivation and treatment

Oocytes were acquired from at least 6 week old CD1 mice. The females were stimulated 46 h prior to oocyte isolation using 5 IU of pregnant mare serum gonadotropin (PMSG; Folligon; Merck Animal Health) per mouse. All animal experiments were performed in accordance to guidelines and protocols approved by Laboratory of Biochemistry and Molecular Biology of Germ Cells at the Institute of Animal Physiology and Genetics in Czech Republic. All animal work was conducted according to Act No. 246/1992 on the protection of animals against cruelty, issued by experimental project #215/2011, certificate #CZ02389, issued by the Ministry of Agriculture. Fully grown NE oocytes were isolated into transfer medium (TM)^72^ supplemented with 100 µM 3-isobutyl-1-methylxanthine (IBMX; Sigma Aldrich) for prevention of spontaneous meiotic resumption. Selected oocytes were freed of cumulus cells and cultivated in M16 medium (Millipore) without IBMX at 37 °C, 5% CO_2_ for 0 (NE), 3 (NEBD), 7 (MI) or 12 h (MII).

### PCR and qPCR

RNeasy Plus Micro kit (Qiagen) was used for RNA extraction following the manufacturer’s instructions. Reverse transcription was performed with Sensiscript RT kit (Qiagen) using random hexamers and oligo(dT) primers. PCR was performed with PPP master mix (TOP-Bio). Following program was used: 94 °C 1 min; 94 °C 18 sec; 58 °C 15 sec; 72 °C. Products were separated on 1.2% agarose gel with GelRed (41003, Biotinum) staining. Presented images were cropped from membranes, contrast and brightness was adjusted using Adobe Photoshop CS3. Full images of segments are shown in Suppl. Fig. 5.

The expression of selected genes was measured using QIAGEN OneStep RT-PCR Kit (Qiagen) with SybrGreen I (Molecular Probes, Eugene) in real-time detection. Measurements were carried out on a RotorGene 3000 cycler (Corbett Research, Australia). The relative concentrations of templates in different samples were determined in Corbett Research software by comparative analysis. The relative internal standard *Gapdh* was used for normalization of the qPCR results. Used primers are listed in Supplementary Table 1A.

### RNA FISH

Fixed oocytes (15 min in 4% PFA were pre-treated with protease III provided in RNAScope H_2_O_2_ and Protease Reagents kit (diluted 1:15 in nuclease free water; Cat. No. 322381, ACD) for 10 min. Each sample was then incubated with RNAScope probes (Supplementary Table 1B) to detect *Ankyrin2.3*, *Neat2*, positive control (*Polr2a*), and negative control (*DapB*) 2 h in 40 °C. RNAScope protocol for amplification was followed using reagents in RNAScope Multiplex Fluorescent Detection Reagents v2 kit (Cat. No. 323110, ACD), with extended washing: 2 × 10 min washing after probe hybridization (1x wash buffer diluted in nuclease free water; RNAScope Wash Buffer Reagents, Cat. No. 310091, ACD); v2 Amp1 30 min, 40 °C, 2 × 5 min 1x wash buffer; v2 Amp2 30 min, 40 °C, 2 × 5 min 1x wash buffer; v2 Amp3 15 min, 40 °C, 2 × 5 min 1x wash buffer. After amplification, HRP-C1/C2/C3 was used on the corresponding channels of specific probes, for 15 min, 40 °C. Oocytes were washed again 2 × 5 min in 1x wash buffer. TSA Cy5 dye (Perkin Elmer) diluted to 1:1500 in TSA buffer (ACD) was used for fluorescent labeling of the amplified signal. After washing and application of HRP blocker (30 min in 40 °C), samples were washed a final time 2 × 5 min in 1x wash buffer and mounted in Prolong Gold Antifade Mountant with DAPI (Life Technologies) on epoxy coated slides (Thermo Scientific). Images were obtained using a confocal microscope (Leica SP5). Image quantification was performed using ImageJ software (http://rsbweb.nih.gov/ij/).

### Ribopuromycilation and proximity ligation assay and (Puro-PLA; PLA)

Oocytes were cultivated with or without inhibitors: 1 h with 100 µM cycloheximide (CHX; Sigma Aldrich), 1 h with 100 µM 4EGI-1 (Bio Techne), or 10 min with 0.5 µM EDTA^73^ and then fixed 15 min in 4% PFA and permeabilized (10 min in 0.1% Triton X-100 in PBS). For ribopuromycilation oocytes were incubated 15 min with 1 µM puromycin in M16 prior fixation. The cells were then incubated with primary antibodies: anti-RPL24 (1:150; PAS-62450, Thermo Fisher), anti-RPS6 (1:150; 74459, CST); mouse anti-puromycin (1:200; MABE343, Millipore) and rabbit Ankyrin B (1:100; sc-28560; Santa Cruz) at 4 °C overnight. For negative control only anti-puromycin or anti-RPL24 antibody was used. PLA was performed following instructions of PLA Duolink kits (DUO92006 and DUO92008, Sigma Aldrich) and a previously published protocol^15^. The samples were washed using PBS and subsequently in Wash Buffer A (Sigma Aldrich) and incubated with 40 μL of the probes reaction mix for 1 h at 37 °C. Next the samples were washed in 1x Wash Buffer A for 2 × 5 min and the following ligation reaction was performed for 30 min at 37 °C. After washing (2 × 5 min) in Wash Buffer A, 40 μL of amplification reaction reagent was added to each sample and incubated for 100 min at 37 °C. The samples were then washed for 2 × 10 min in 1x Wash Buffer B (Sigma Aldrich) and for 2 min in 0.01x Wash Buffer B, and mounted on a slide using Vectashield Mounting Medium with DAPI (H-1500, Vector Laboratories). Inverted confocal microscope (Leica SP5) was used for sample visualization. Image quantification and assembly were performed using ImageJ and Adobe Photoshop CS3. Spindle area was defined by DAPI staining and number of interactions was analyzed in ImageJ software (http://rsbweb.nih.gov/ij/).

### Immunocytochemistry

Fixed oocytes (15 min in 4% PFA; Sigma Aldrich) were permeabilized in 0.1% Triton X-100 for 10 min, washed in PBS supplemented with polyvinyl alcohol (PVA, Sigma Aldrich) and incubated in primary antibodies overnight at 4 °C. The following primary antibodies diluted in PVA/PBS were used: rabbit Ankyrin B (1:150; sc-28560, Santa Cruz), goat Ankyrin B (1:150; sc-14995, Santa Cruz), mouse monoclonal anti-acetylated tubulin (1:150; T6793, Sigma Aldrich), rabbit 4E-BP1(Thr70) (1:500; cs-9455S, CST), rabbit PABP (1:150; sc-28834). Oocytes were then washed 2 × 15 min in PVA/PBS and primary antibodies were detected using relevant Alexa Fluor 488/594/647 conjugates (Invitrogen) diluted to 1:250 for 1 h at room temperature. Washed oocytes (2 × 15 min in PVA/PBS) were mounted onto slides using Vectashield Mounting Medium with DAPI (H-1500, Vector Laboratories). Inverted confocal microscope (Leica SP5) was used for sample visualization. Image quantification and assembly were performed using ImageJ and Adobe Photoshop CS3. Experiments were repeated 3x with 20–30 oocytes per group/experiment.

### Microinjection of oocytes and live-cell imaging

Isolated NE oocytes were microinjected in TM with IBMX using inverted microscope Leica DMI 6000B, TransferMan NK2 (Eppendorf) and FemtoJet (Eppendorf). Solution used for oocyte injection contained: 20 ng/µL of *in vitro* transcribed *H2b:gfp* RNA (mMessage, Ambion) from plasmid (provided by Dr. Martin Anger, Laboratory of Cell Division Control, IAPG CAS) in combination with 1 mM morpholinos (control: CCTCTTACCTCAGTTACAATTTATA and *Ank2.3*: TGTGACTGCCTGTCTACCATCAAAC; Gene Tools) - to block translation of *Ank2.3* mRNA. 1.5 h after microinjection oocytes were washed from IBMX and cultivated to MII stage. Microinjected oocytes were placed into 4-well culture chamber (Sarstedt) in 10 µL of equilibrated M16 media (37.5 °C, 5% CO_2_) covered with mineral oil (M8410; Sigma Aldrich). The cells were imaged using inverted microscope Leica DMI 6000B equipped with a controlled chamber system (Tempcontroller 2000–2 Pecon, and a CO_2_ controller, Pecon). Time lapse movies (LAS X, Leica microsystems) of meiotic maturation of microinjected oocytes with chromatin marker *(H2b:gfp*) and morpholinos were used for phenotype evaluation (nuclear envelope breakdown, polar body extrusion).

### Immunoblotting

An exact number of cells (15–30 oocytes) was washed in PVA/PBS and frozen to −80 °C. Prepared samples were lysed in NuPAGE LDS Sample Buffer (NP0007, Thermo Fisher Scientific) and NuPAGE Sample Reducing Agent (NP0004, Thermo Fisher Scientific) and heated at 100 °C for 5 min. Proteins were separated on precast gradient 4–12% SDS–PAGE gel (Thermo Fisher Scientific) and blotted to Immobilon P membrane (Millipore) in a semidry blotting system (Biometra GmbH) at 5 mA cm^−2^ for 25 min. Membranes were then blocked in 5% skimmed milk dissolved in 0.05% Tween-Tris buffer saline (TTBS), pH 7.4 for 1 h. Membranes were incubated overnight at 4 °C with the following antibodies diluted in 1% milk/TTBS: rabbit Ankyrin B (1:500; sc-28560, Santa Cruz) and rabbit GAPDH (1:30 000; G9545, Sigma Aldrich). Secondary antibodies with Peroxidase were used (711-035-152Anti-Rabbit Donkey, or 715-035-151 Anti-Mouse Donkey, both Jackson ImmunoResearch), diluted 1:7500 in 1% milk/TTBS for 1 h at room temperature. ECL (Amersham) was used for visualization of immunodetected proteins on X-ray films. The films were scanned by calibrated densitometer (GS-800, Bio-Rad Laboratories) and quantified in ImageJ. Presented images were cropped from membranes, contrast and brightness was adjusted using Adobe Photoshop CS3. Full images of segments are shown in Suppl. Fig. 5.

### Mfold analysis

We used FASTA sequence *Ank2.3* mRNA for analysis by Mfold software (version 2.3 energies; http://unafold.rna.albany.edu).

### Statistical analysis

Mean and standard deviation values were calculated in MS Excel, Student’s *t*-test was used to determine the statistical significance of the differences between groups and *p < 0.05 was considered as statistically significant (in graphs labeled with a star). Other p-values were distinguished: **p < 0.01 and ***p < 0.001. Non-significant statistical results are designated with “NS”.

## Supplementary information

Supplementay info
