# Publisher Correction: Spatio-temporal expression of ANK2 promotes cytokinesis in oocytes

**DOI:** 10.1038/s41598-020-60116-0

**Published:** 2020-02-18

**Authors:** Anna Tetkova, Denisa Jansova, Andrej Susor

**Affiliations:** 1 Laboratory of Biochemistry and Molecular Biology of Germ Cells, IAPG CAS, Libechov, Czech Republic; 2 Department of Cell Biology, Faculty of Science, Charles University, Prague, Czech Republic

Correction to: *Scientific Reports* 10.1038/s41598-019-49483-5, published online 11 September 2019

The Acknowledgements section in this Article is incomplete.

“We thank Jaroslava Supolikova and Marketa Hancova for assistance with experiments and Martin Anger for kindly providing the H2B:gfp plasmid. We also thank to Lenka Gahurova, Rajan Iyyappan and Chih-Jen Lin for critical reading of the manuscript.”

should read:

“We thank Jaroslava Supolikova and Marketa Hancova for assistance with experiments and Martin Anger for kindly providing the H2B:gfp plasmid. We also thank to Lenka Gahurova, Rajan Iyyappan and Chih-Jen Lin for critical reading of the manuscript. The authors declare no competing interests and no financial interests. This research was funded by MSMT (EXCELLENCECZ.02.1.01/0.0/0.0/15_003/0000460 OP RDE), GACR (18-19395S) and GA UK (1570218).”

