# Author Correction: Spatio-temporal expression of ANK2 promotes cytokinesis in oocytes

**DOI:** 10.1038/s41598-020-65541-9

**Published:** 2020-05-19

**Authors:** Anna Tetkova, Denisa Jansova, Andrej Susor

**Affiliations:** 1 Laboratory of Biochemistry and Molecular Biology of Germ Cells, IAPG CAS, Libechov, Czech Republic; 2 Department of Cell Biology, Faculty of Science, Charles University, Prague, Czech Republic

Correction to: *Scientific Reports* 10.1038/s41598-019-49483-5, published online 11 September 2019

This Article contains errors.

As a result of an error during figure assembly, in Figure 5A the images corresponding to CHX are duplicates of images for NTC. Correct Figure 5A is shown below as Figure 1.

**Figure 1:**
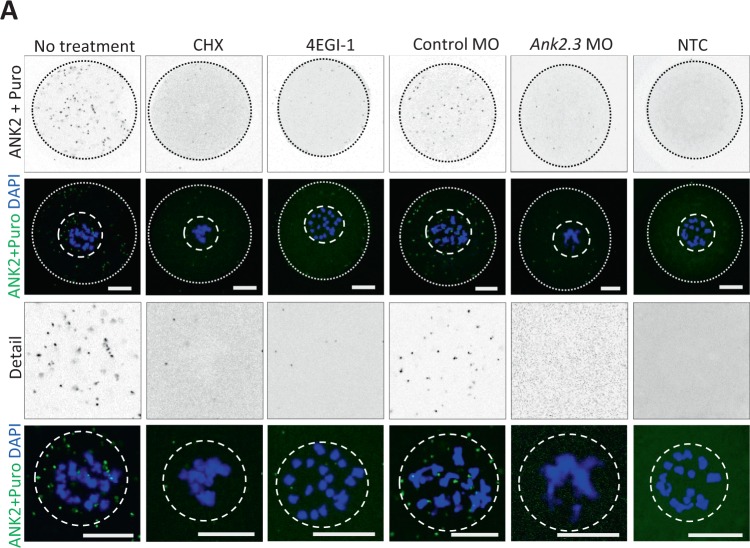
.

These corrections do not affect the conclusions of the Article.

