# Supplementary material for: Spatio-temporal expression of ANK2 promotes cytokinesis in oocytes
Source: Sci Rep. 2019 Sep 11;9:13121. doi: 10.1038/s41598-019-49483-5 (PMC6739377; doi:10.1038/s41598-019-49483-5)
Supplement: Supplementary file 1 — Supplementay info [file 41598_2019_49483_MOESM1_ESM.pdf]

SUPPLEMENTAL FIGURES

Spatio-temporal expression of ANK2 promotes cytokinesis in oocytes

Anna Tetkova, Denisa Jansova, Andrej Susor*

Laboratory of Biochemistry and Molecular Biology of Germ Cells, IAPG, Czech Republic

*susor@iapg.cas.cz

***Suppl. Fig. 1: Ank2.3* mRNA is germ cell specific. A**) Confocal RNA FISH images detecting *Ank2.3* mRNA (grey, red) in oocytes and cumulus cells. DNA in blue (DAPI). Representative images from three biological experiments (n≥19). Scale bar 20 µm. **B**) Quantification of *Ank2.3* mRNA foci in oocytes and cumulus cells. N=46; Student's *t*-test, *** p<0.001; mean ±SD. **C**) Representative images of RNA FISH detecting *DapB* bacterial RNA (Bacillus subtilis, str. SMY) RNA (EF191515.1) which serves as a negative control. Representative images from at least three biological experiments. Scale bar 20 µm. **D**) Quantification of negative control *DapB* RNA foci in oocytes. Student's *t*-test, mean ±SD (1.25; 1.3); NS non-significant; n≥20.

***Suppl. Fig. 2: Neat2* RNA become dramatically degraded post NEBD. A**) qRT-PCR mRNA expression of *Gapdh* and *Neat2* in NE and MII oocytes. Data from three independent experiments were normalized to NE oocytes. Student's *t*-test, *P<0.05; **P<0.01; ***P<0.001; NS non-significant. SD ±0.064; 0.021. **B**) Number of RNA foci at the equatorial confocal section from RNA FISH. N≥18, Student's *t*-test; **P<0.01; NS non-significant.

***Suppl. Fig. 3:* Ank2.3 mRNA contains oligopyrimidine tract at 5'UTR.** Secondary structure of *Ank2.3* mRNA generated by mFold software[40].

***Suppl. Fig. 4*: Localization and expression of ANK2 protein during meiotic progression of oocyte. A**) Immunolabeling of endogenous ANK2 (grey and green) and tubulin (grey and red) in NE (0h), NEBD (3h), MI (7h) and MII (12h) oocytes. Representative images from at least three biological replicates; n≥10. Scale bars 20µm. **B**) Detail of ANK2 localization in the oocyte bipolar spindle. Arrowhead depicts cytoplasmic protrusion. Scale bar 10µm.

***Suppl. Fig. 5*: Full gel and immunoblots of segments shown in the main Figures 1A, 6B and 7B**. Arrows denote the bands used.

***Supplementary Table 1*: Supplementary tables of primers and RNA FISH probes. A**) Primers designed for PCR and qRT-PCR. B) Probes used in RNA FISH (RNAScope).

***Suppl. Fig. 5*: Full images of gels and immunoblots of segments shown in the main Figure 1, Figure 6 and Figure 7.**

Supplementary Fig. 1

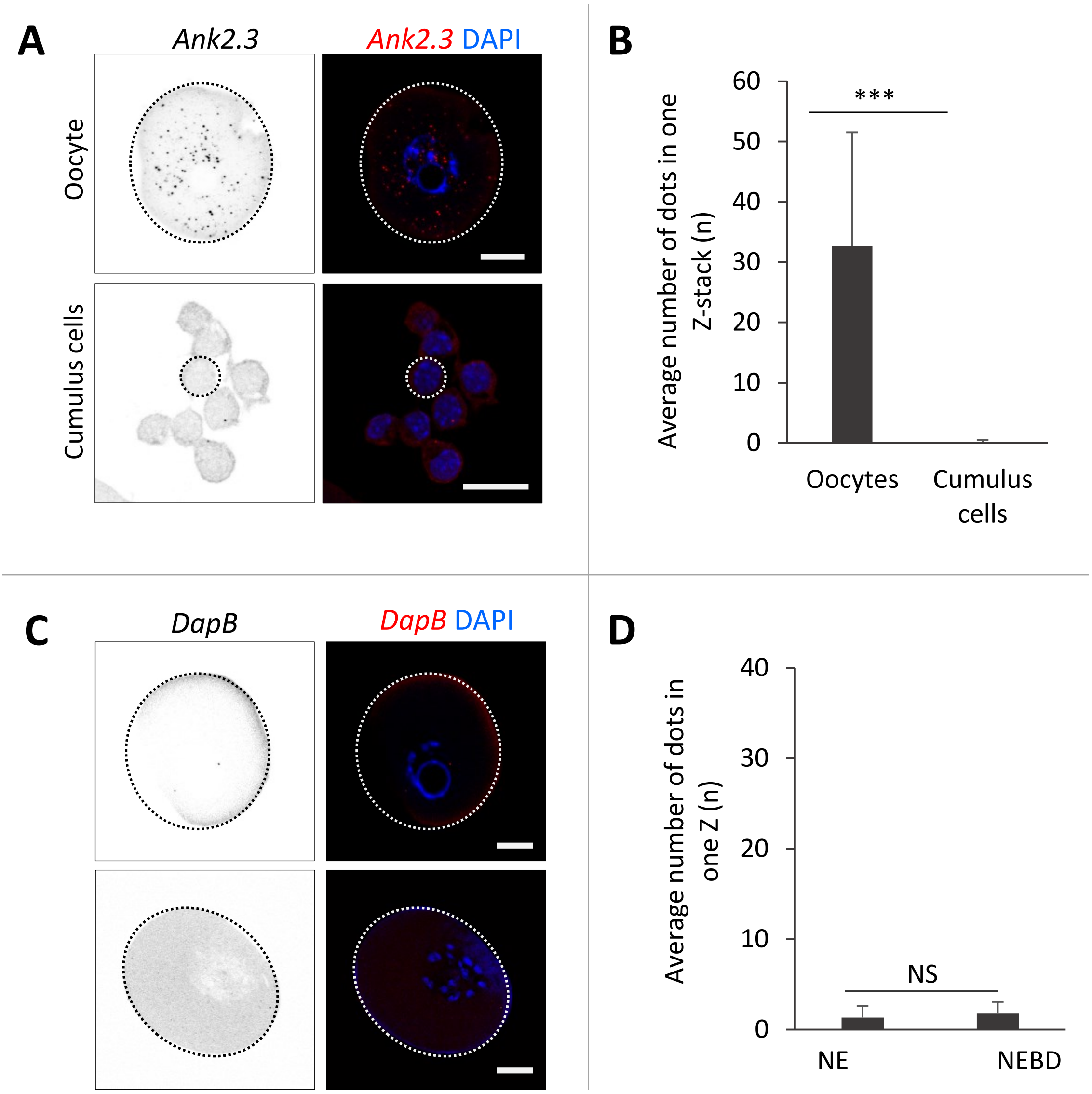

Supplementary Fig. 2

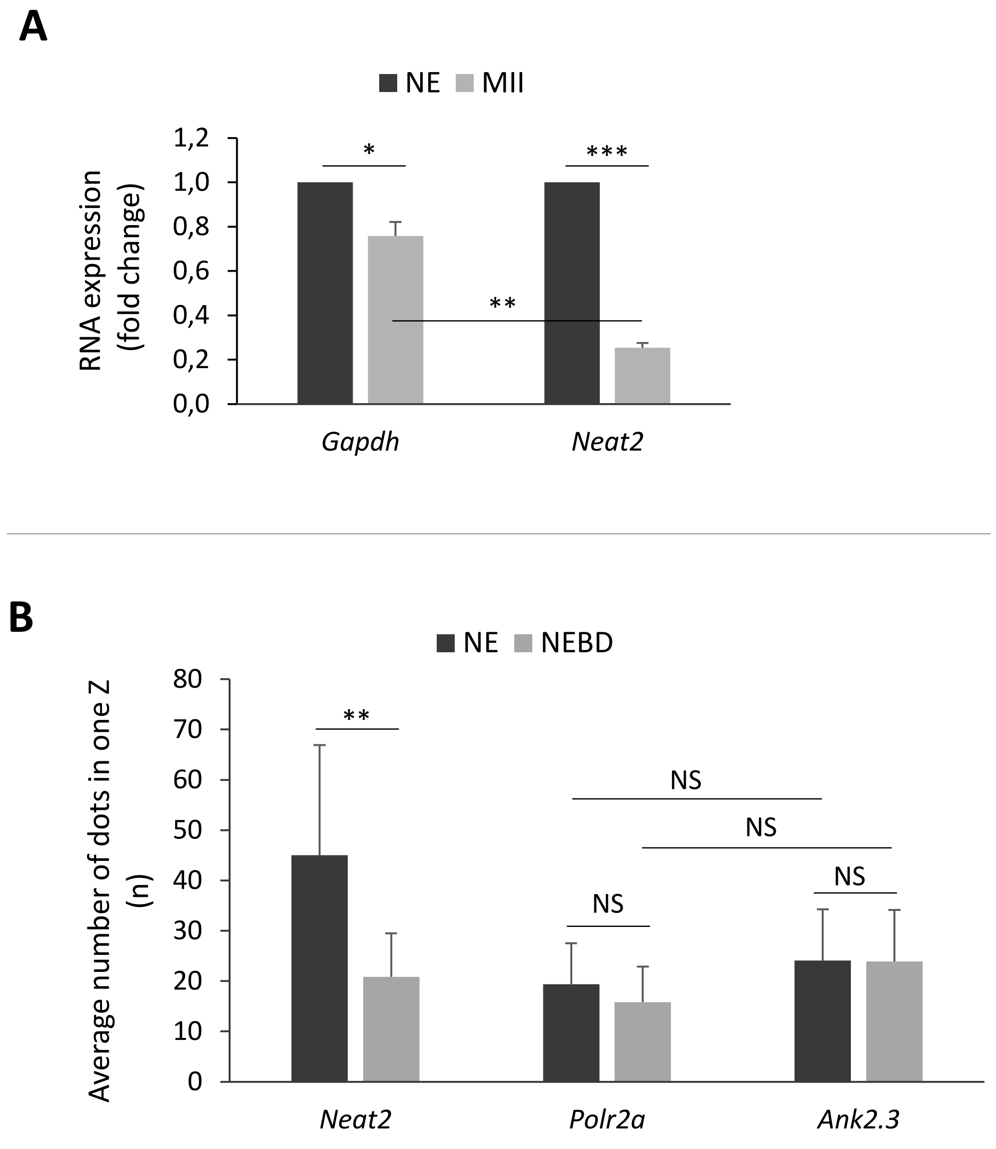

Supplementary Fig. 3

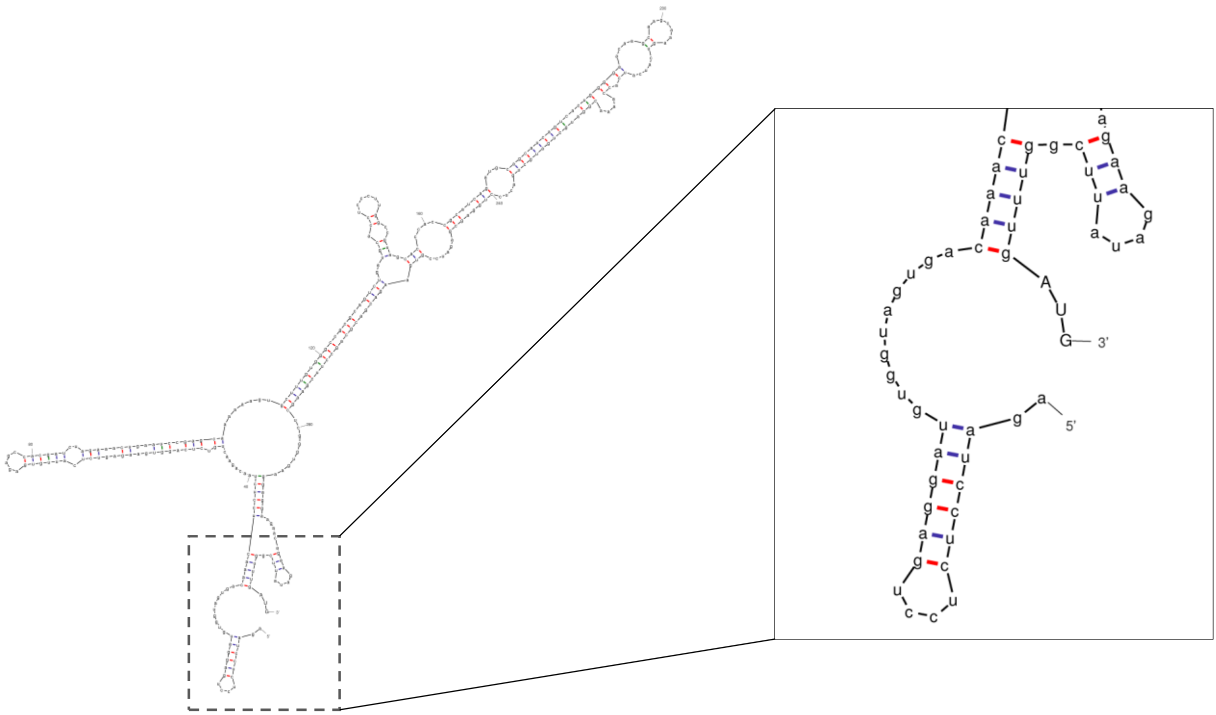

Supplementary Fig. 4

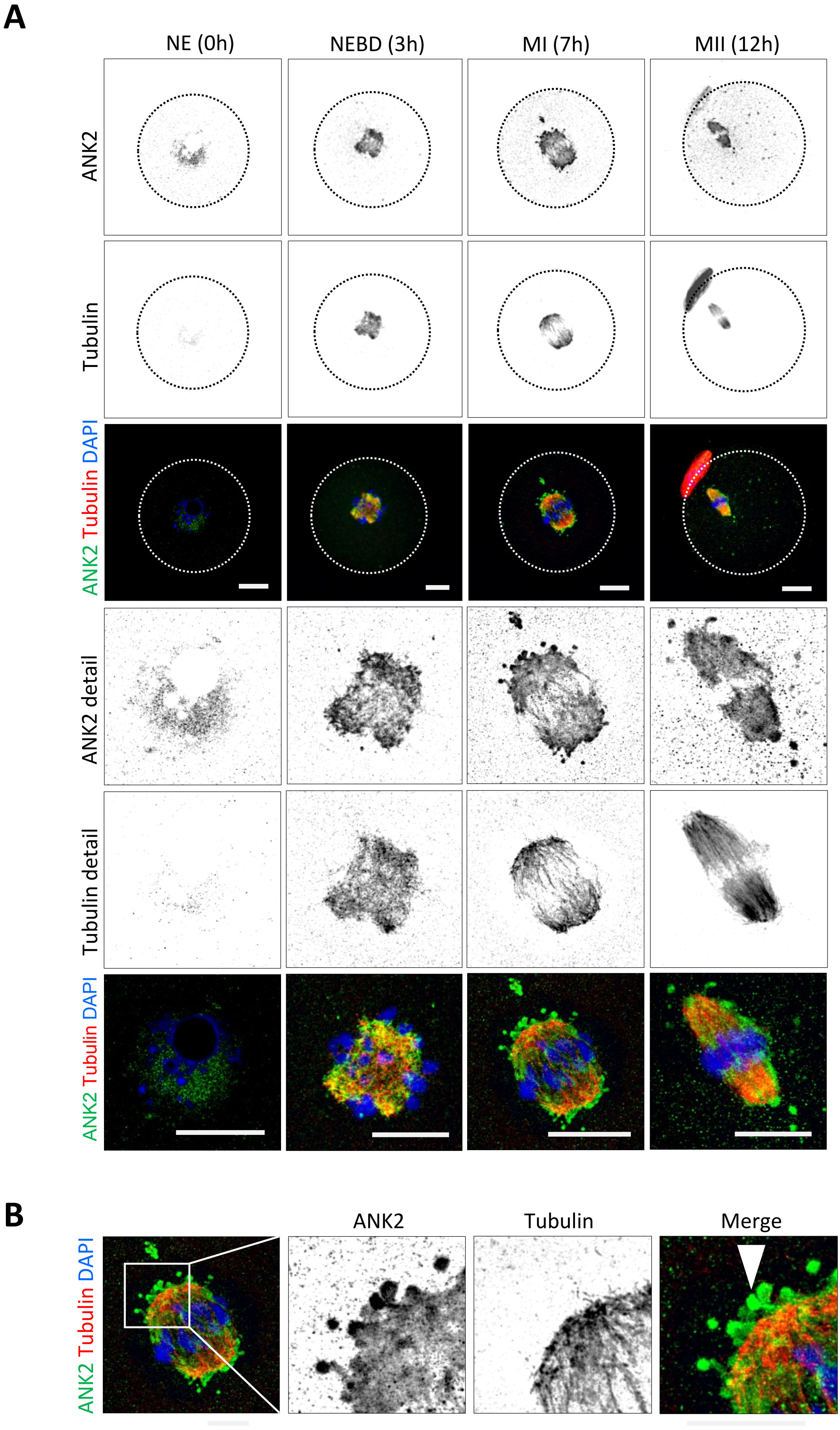

Supplementary Fig. 5

A

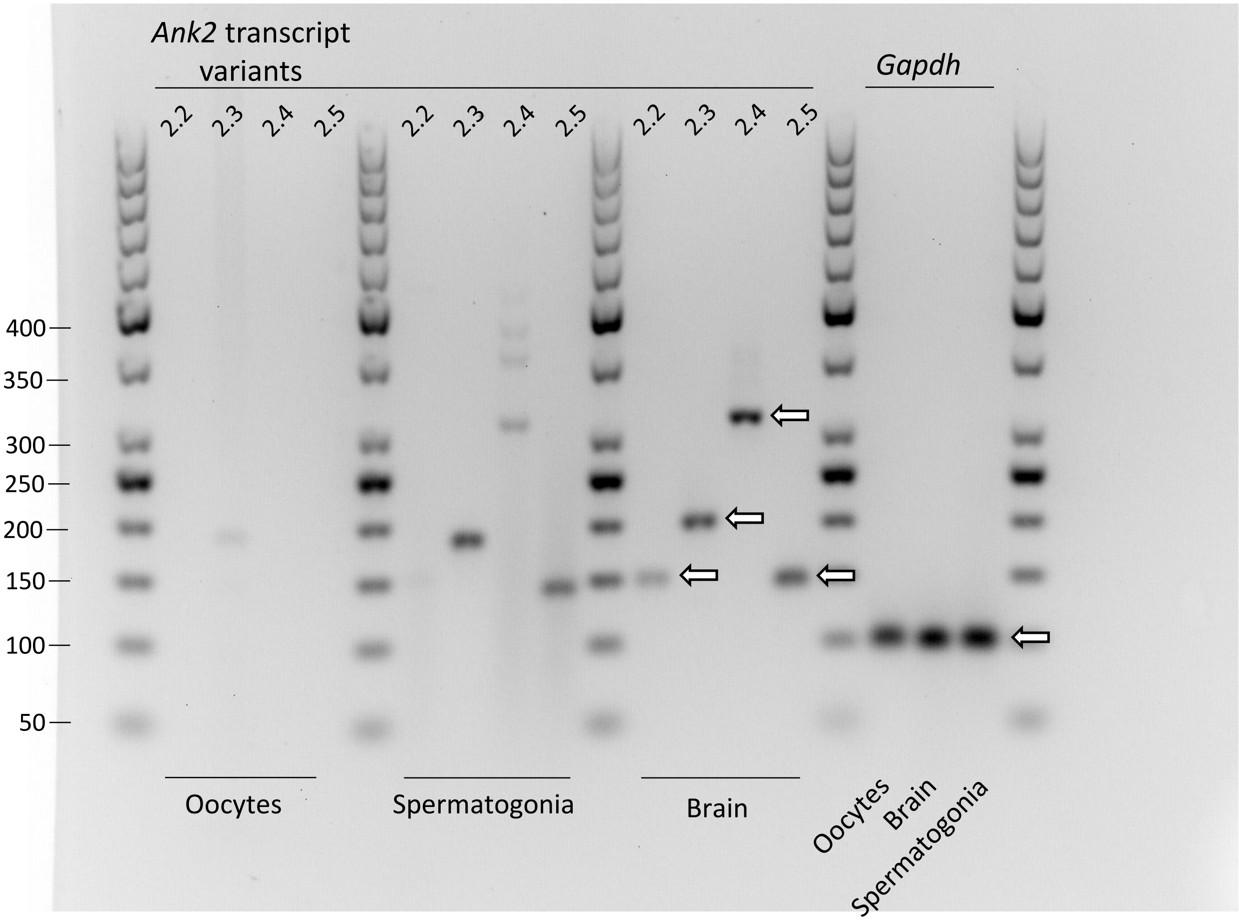

B

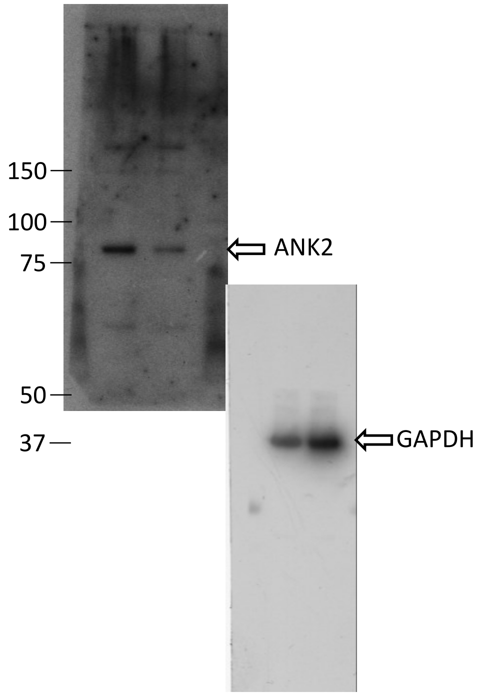

C

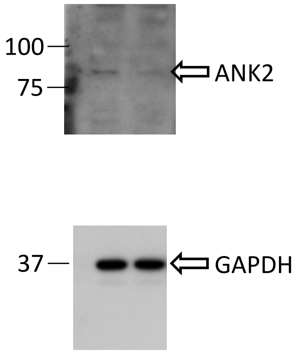

Supplementary Table. 1

A

Official symbol (gene)	Gene bank ID	Forward (5' - 3')	Reverse (5' - 3')	Product size (bp)	Annealing temperature (°C)
Ank2 transcript variant 2	NM_178655	GCAGATGGCCTG ACTCTTGA	TGCCATCCAGGAAC TGACTG	293	60
Ank2 transcript variant 3	NM_001034168.1	GCATCAGTCACT GGGGAACA	GGGTCCTAGCAGG AGTGGTA	196	60
Ank2 transcript variant 4	NM_001327938.1	CTGCGGTCGCCT AGAAGC	TGAGTCCATTCTGA TTGCAGGTA	309	60
Ank2 transcript variant 5	NM_001327939.1	AGGCTGTGATGG GAAAGTCG	CAGCAGCTTCTCTC AGCGAT	137	60
Gapdh	XM_001476707.3	CGGGAAGCCCAT CACGATTT	GGTCATGAGCCCTT CCACAA	93	58
Neat2	NR_002847.2	AGGGAAAAGGGG GAAAGC	AGGGGTGAAGGGTC TGTGAT	133	58

B

Gene	Accession No.	Target Region	No. of ZZ pairs	Cat. No.
Ank2	NM_001034168.1	2685 - 3609	20	413221
DapB	EF191515	414 - 862	10	310043
Neat2	NR_002847.2	712 - 2338	30	313391
Polr2a	NM_009089.2	2802 - 3678	20	312471
